# Systemic surveillance for uveal melanoma: Approach to optimization

**DOI:** 10.1371/journal.pone.0355405

**Published:** 2026-08-19

**Authors:** Emily C. Zabor, Yağmur Seda Yeşiltaş, Arun D. Singh

**Affiliations:** 1 Department of Quantitative Health Sciences, Cleveland Clinic Research, Cleveland, Ohio, United States of America; 2 Department of Ophthalmology, University of Health Sciences, Gülhane Medical Faculty, Ankara, Turkey; 3 Department of Ophthalmic Oncology, Cole Eye Institute, Cleveland, Ohio, United States of America; University of Rochester FEI: University of Rochester David and Ilene Flaum Eye Institute, UNITED STATES OF AMERICA

## Abstract

**Importance:**

There is lack of evidence assessing surveillance protocols to detect metastasis in patients with uveal melanoma (UM).

**Objective:**

To explore hypothetical surveillance schedules anchored in the real-world data of our patient cohort with known prognostication status wherein, surveillance intervals ranging from every 1-month to every 12-months were statistically analysed.

**Design:**

Retrospective, cohort study.

**Setting:**

Single center.

**Participants:**

Patients clinically diagnosed with UM and managed with standard of care local therapy who underwent prognostication by commercially available Gene Expression Profile (GEP) (Castle Biosciences, Friendswood, Texas, USA). Those classified as having low risk (Class 1) were offered a standard protocol (SP; hepatic ultrasonography (US) at 6-month intervals) while those with high risk (Class 2) were recommended to undergo enhanced protocol (EP) that incorporated either higher frequency (US every 3 months) or enhanced modality (EM, hepatic computed tomography/magnetic resonance imaging).

**Main outcome Measures:**

Optimal scan interval.

**Results:**

349 patients with median age of 62 years and 54% were male. Most patients were Class 1 (65%) and had choroidal location (75%). Median follow-up among survivors was 30.9 months (IQR: 19–57.9). 67 patients developed metastasis. The 5-year overall survival (OS) was 80% and the 5-year metastasis-free survival (MFS) was 71%. For Class 1 patients, 12-month scan interval was optimal as it had the minimal Euclidean distance to the point (0, 0) with a median of 2.92 months from negative to positive scan with an average of 2.83 scans per patient. For Class 2 patients, a 9-month scan interval was optimal as it minimized the Euclidean distance to the point (0, 0) with an average of 3.09 scans per patients and a median of 2.93 months from negative to positive scans. There was an association between the longer scan interval and size of metastatic lesion (>=6 months versus <6 months, p = 0.002).

**Conclusions and relevance:**

Our analysis suggests that the frequency of hepatic imaging for detection of metastasis can be tailored on the risk status to 12 months and 9 months for patients with low and high-risk tumors, respectively. Our study provides a framework for future studies assessing surveillance protocol designs, efficacy, and impact on patient survival.

## Introduction

In patients with uveal melanoma (UM), metastasis is generally not detectable at ocular presentation, with staging evaluation negative in the vast majority of cases [1]. Although micro metastasis occurs early in UM [2,3], metastases become detectable by imaging modalities within the first 5 years followed by a gradual decline over the next 10 years. Death due to melanoma is rare beyond 20 years [4]. Surveillance for metastasis, therefore, is an integral and important aspect of overall long-term management of the patient. As liver is the predominant site (93%), liver directed imaging is the hallmark of surveillance protocols [5].

Assessment of prognostic factors of UM allows for risk stratification for metastasis [6,7], which has led to risk-stratified surveillance strategies [8,9]. We have previously reported on the efficacy of surveillance protocols in patients with low risk [10] and high risk UM [11]. The vast majority of patients with low risk UM did not develop metastasis by 5 years (96%).^10^ Surveillance protocols in such patients had very low yield and their impact on survival could not be assessed. In patients with high risk UM, while enhanced-modality surveillance detected smaller hepatic metastatic lesions than standard protocol, this did not translate into improved overall survival [11].

While a number of National or Consensus Guidelines exist including those from the American Society of Clinical Oncology (ASCO) [12], Polish Society of Oncology [13], Scotland [14], UK [15], Canada [16] and the NCCN [17], none are supported by evidence for their recommendations (**Table 1**). The lack of evidence became apparent in a recently conducted systematic review [18]. Despite exhaustive search of the literature covering a period of 15 years (2010–2024), the authors could find only 12 studies [11,19–29]. that were suitable for inclusion. Due to the heterogeneity of studies, meta-analysis could not be done. All studies were assessed to be of poor quality by mNOS scoring [30] except one by Yesiltas et al., [11] which was rated as fair. Recently conducted Delphi panel highlighted the lack of evidence assessing surveillance protocols [31].

**Table 1:** Surveillance of patients with uveal melanoma: Summary of institutional guidelines.

Institution	Author	Year	Risk	Liver imaging modality	Frequency (months)	Duration (years)
**ASCO** ^**12**^	Francis	2013	High	MRI/ CT/ PET	3-6 months	–
			Low	Not specified	6-12 months	–
**Polish Society of Oncology** ^**13**^	Rutkowski	2022	High	MRI (preferred) or US ± CT CAP or PET	3-6 months	0-5 years
					6-12 months	6-10 years
			Medium	Not specified	6–12 months	Not specified
			Low	If indicated	Not specified	Not specified
**Scotland** ^**14**^	Chadha	2023	High	MRI	6 months	10 years
			Low	US	6 months	10 years
**UK** ^**15**^	Carter	2023	High	Not specified	6 months	Lifelong
**Alberta** ^**16**^ **(Canada)**	Weis	2023	High	MRI/US alternating	6 months	10 years
			Low	US	12 months	10 years
**NCCN** ^**17**^ **(USA)**		2024	High	MRI or US ± CT CAP or CXR	3-6 months	0-5 years
					6-12 months	6-10 years
			Medium		6–12 months	10 years
			Low		12 months	5 years

ASCO: American Society of Clinical Oncology; CT: Computed tomography; CT-CAP: Computed tomography scanning of the chest, abdomen and pelvis; CXR: Chest X-ray; MRI: Magnetic resonance imaging; NCCN: National Comprehensive Cancer Network; PET: Positron emission tomography; UK: United Kingdom; US: Ultrasonography; USA: United States of America.

In the present study, we have performed statistical analyses using hypothetical surveillance schedules anchored in the real-world data of our patient cohort with known prognostication status. The goal was to compare surveillance intervals ranging from every 1-month to every 12-months to provide real-world evidence-based guidance on surveillance frequency in UM patients.

## Methods

### Study design

This study was approved by the Cleveland Clinic Foundation Institutional Review Board (IRB #:23–268). The ethics committee waived the requirement for informed consent. The study adhered to the tenets of the Declaration of Helsinki. The data for this retrospective study was collected between April 2023 to Dec 2024. One study site covered the whole study period. Patients were treated for primary uveal melanoma with ocular therapy including enucleation, plaque brachytherapy or primary resection following standard of care. In addition, baseline systemic staging that included a CT scan of the chest, abdomen and pelvis with and without contrast was advised in all cases prior to ocular therapy and prognostication.

### Prognostication

It is institutional practice to recommend molecular prognostication with GEP using the only commercially available GEP assay (Castle Biosciences, Friendswood, Texas, USA). Prognostic Class is determined by GEP testing of a tumor sample obtained using a transscleral or transvitreal fine needle aspiration biopsy technique if a patient elects to undergo prognostic testing. For patients undergoing treatment with plaque brachytherapy, fine needle aspiration biopsy is performed at the time of plaque insertion. Based upon prognostication, patients were categorized into one of three groups: low risk for metastasis (Class 1), high risk for metastasis (Class 2), and unknown risk for metastasis (Class X). Patients with known prognostication as either low risk (Class 1) or high risk (Class 2) were included in the primary analysis. Sensitivity analysis was performed in a separate cohort of patients with unknown risk (Class X). To identify a subset of Class X patients to conduct sensitivity analysis, we matched Class X patients to patients with known class to ensure patients with a variety of features were included. Matching was done using nearest neighbor matching based on tumor location and thickness. In this way, of the original 169 Class X eyes, 40 were matched to Class 2 patients and 20 were matched to Class 1 patients.

### Surveillance protocols

Patients classified as having low risk (Class 1) are offered a standard protocol (SP; hepatic ultrasonography (US) at 6-month intervals) [10] while those with high risk (Class 2) are recommended to undergo enhanced protocol (EP) that incorporates either higher frequency (US every 3 months) or enhanced modality (EM, hepatic computed tomography/magnetic resonance imaging) [10]. Those that could not be prognosticated (Class X) because of patient preference, small tumor size, macular tumor location, monocular status, or presence of co morbidities underwent surveillance using standard protocol [9]

### Demographics

Patient and disease characteristics were summarized using the median and first and third quartiles when continuous, and the number and percentage when categorical.

### Survival

Metastasis-free survival (MFS) was defined from the date of primary ocular treatment to the date when metastasis was first detected. Patients without metastasis were censored at their last scan date. Overall survival (OS) was defined from date of primary ocular treatment to the date of death. Patient still alive were censored at their last contact date.

### Hypothetical scan intervals

To compare different surveillance protocols, we used the observed time from date of primary ocular treatment to date of detection of metastasis or date of last scan in the patient sample and considered a variety of hypothetical scan intervals for surveillance. The hypothetical scan intervals ranged from a scan every one month to every 12 months, by one-month intervals (i.e., scans every one month, scans every two months, scans every three months, etc). Observed data were limited to the first five years of follow-up. As a result, four patients with metastasis detected more than five years after treatment had their follow-up time truncated at five years and were considered metastasis-free at that time. In addition, 47 patients who had not yet developed metastasis but were followed for more than five years had their follow-up time truncated at five years.

Consider a hypothetical surveillance schedule with scans every *t* months, where *t* can take the values 1, 2, 3, …, 12. For each patient in the observed data, we began at the date of primary ocular treatment (time 0) and consider a scan every *t* months until metastasis was detected, until the end of the observed follow-up period (i.e., date of last scan), or until five years, whichever came first. We did this for each value of *t* for each patient. Only patients who were followed for at least *t* months without metastasis were included in the analysis of each scan interval *t*, so that patients who developed metastasis prior to *t* months, or who were followed without metastasis for less than *t* months, were excluded from the analysis of scan interval *t*. Then, for each value of *t*, we summed the total number of scans done across all patients to determine the average number of scans per patient. The average scans per patient were plotted against the scan interval among all patients. In the subset of patients who developed metastasis, we also calculated the median, minimum, maximum, first quartile, and third quartile of time from the last hypothetical scan to the time of observed metastasis detection for each *t*. In the subset of patients who developed metastasis, we plotted the months from hypothetical negative to observed positive scan according to scan interval.

### Optimal scan interval

A receiver operator curve (ROC)-type analysis was conducted to provide further evidence about the optimal scan interval. For each point on the plot of average scans per patient by median months from hypothetical negative to observed positive scan, we calculated the Euclidean distance to the (0, 0) point. The (0, 0) point represents the point with minimal average scans per patient and minimal median months from hypothetical negative to observed positive scan, so that the scan interval *t* that minimizes this Euclidean distance could be considered an optimal scan interval. Optimization results were repeated with stratification by Class (Low/ High/Unknown).

### Largest metastasis size and number of metastasis

Finally, we dichotomized the observed interval from negative to positive scan in the data, among those who developed metastasis, by <=6 months versus>=6 months. This was an empirical cutpoint derived from the prior analyses. We compared the largest metastasis size and number of metastasis according to these scan interval groups using the Wilcoxon rank-sum test and the Fisher’s exact test, respectively. All analyses were conducted in R software version 4.5, including the tidyverse, survival, gtsummary, ggsurvfit, and patchwork packages.

## Results

### Demographics

The analyzed data sample included 349 patients treated for UM between 2013 and 2024 at the Cleveland Clinic Cole Eye Institute. The median patient age was 62 years, and 54% were male. Most patients were Class 1 (65%) and choroidal location (75%) (**S1 Table**).

### Survival

**Fig 1:**
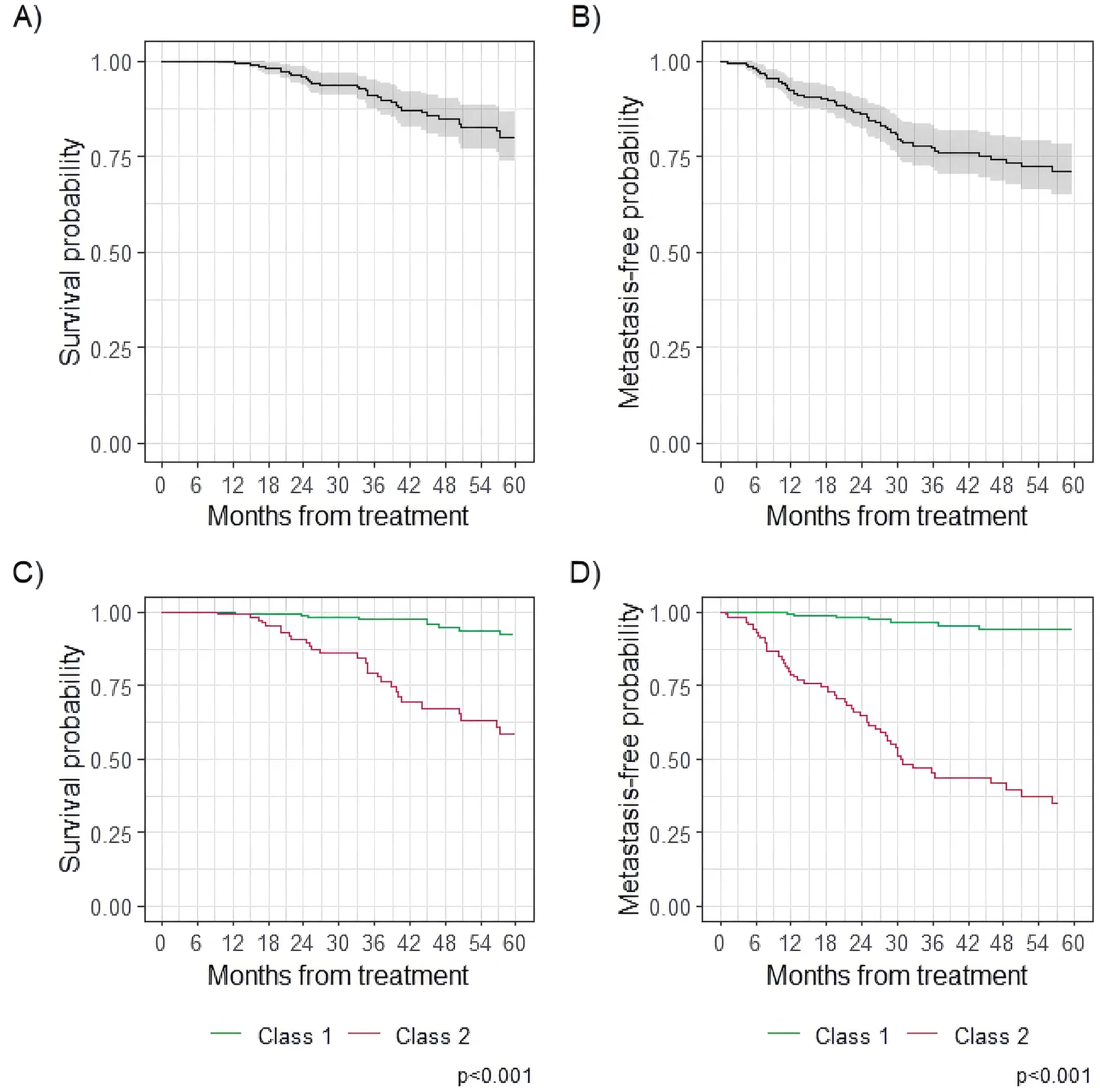
Overall survival (A) and metastasis-free survival (B) in all patients and by class (C – OS; D – MFS).

Median follow-up among survivors was 30.9 months (IQR: 19–57.9). During that time, 48 patients died from any cause. Median follow-up among those without metastasis was 27.8 months (IQR: 13.1–51.1). During that time 67 patients developed metastasis. The 5-year OS was 80%, and the 5-year MFS was 71% (**S2 Table**). OS and MFS were significantly lower for Class 2 patients as compared to Class 1 patients (Fig 1).

### Hypothetical scan intervals

**Fig 2:**
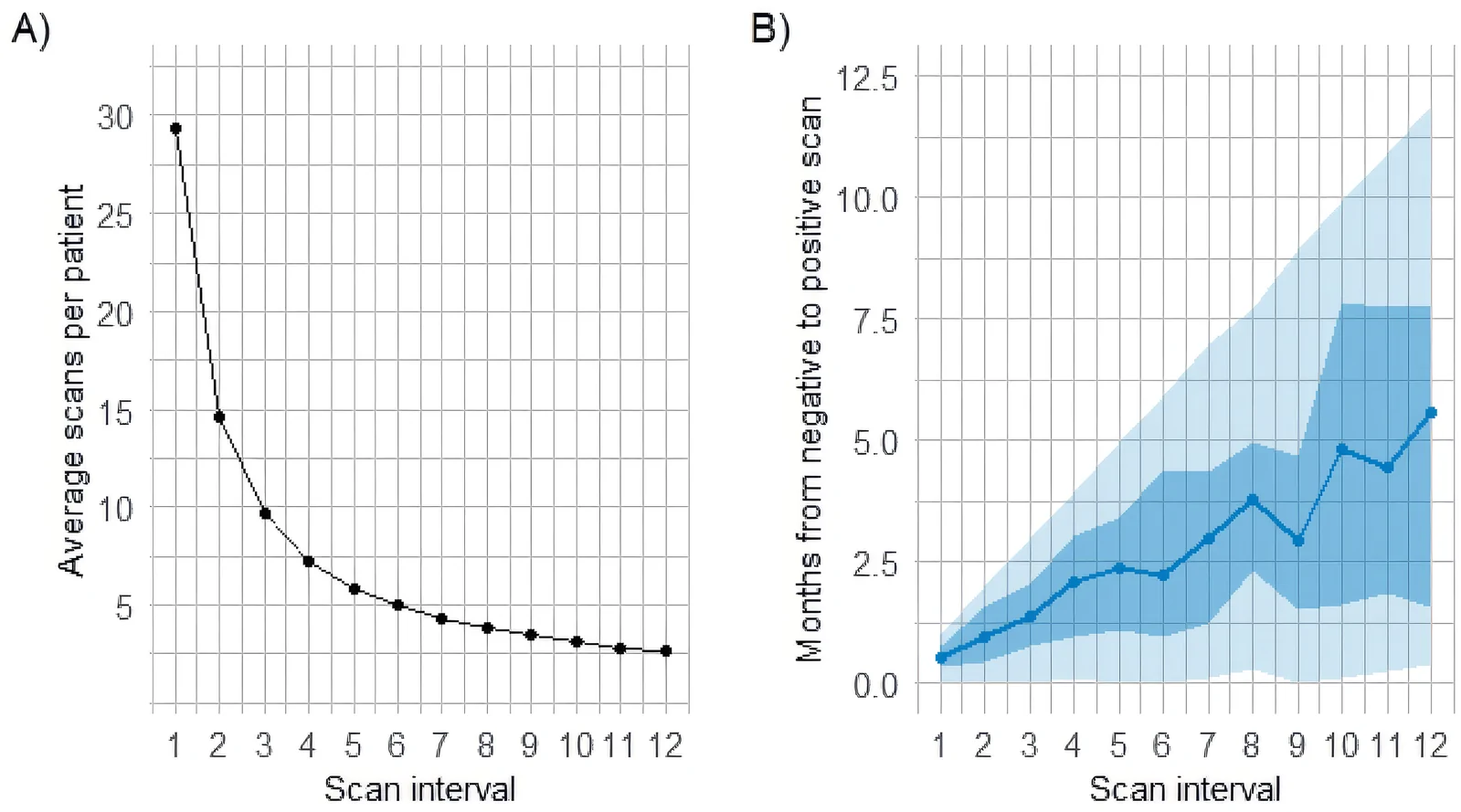
Average number of scans per patient by scan interval among all patients (A) and months from hypothetical negative to observed positive scan by scan interval among patients who developed metases (B), where the solid line represents the median, the dark blue shaded area shows the first to third quartiles, and the light blue shaded area shows the minimum to maximum.

In a hypothetical surveillance schedule, as the scan interval increases from 1 to 12 months, the average number of scans per patient decreases, with the maximum of an average of 29.4 scans per patient at the 1-month scan interval and the minimum of an average of 2.7 scans per patient at the 12-month scan interval (Fig 2A). The 6-month scan interval, which coincides with current practice, is associated with an average of 5 scans per patient. The 6-month scan interval has a median of 2.22 months from negative to positive scan, as compared to 2.35 for the 5-month scan interval and 2.09 for the 4-month scan interval. Depiction of time interval (median months) from a negative to positive scan according to scan interval shows that, on average, a 6-month scan interval is not detecting metastasis much later than a 4-month or 5-month scan interval (Fig 2B). Uncertainty increases as scan interval increases in length, since fewer patients are included as the scan interval increases.

### Optimal scan interval

ROC analysis showed that the 9-month scan interval minimizes the Euclidean distance to the point with 0 average scans per patient and 0 median months from negative to positive scan. The 9-months scan interval leads to an average of 3.45 scans per patient and has a median of 2.93 months from negative to positive scan.

### By GEP class

**Fig 3:**
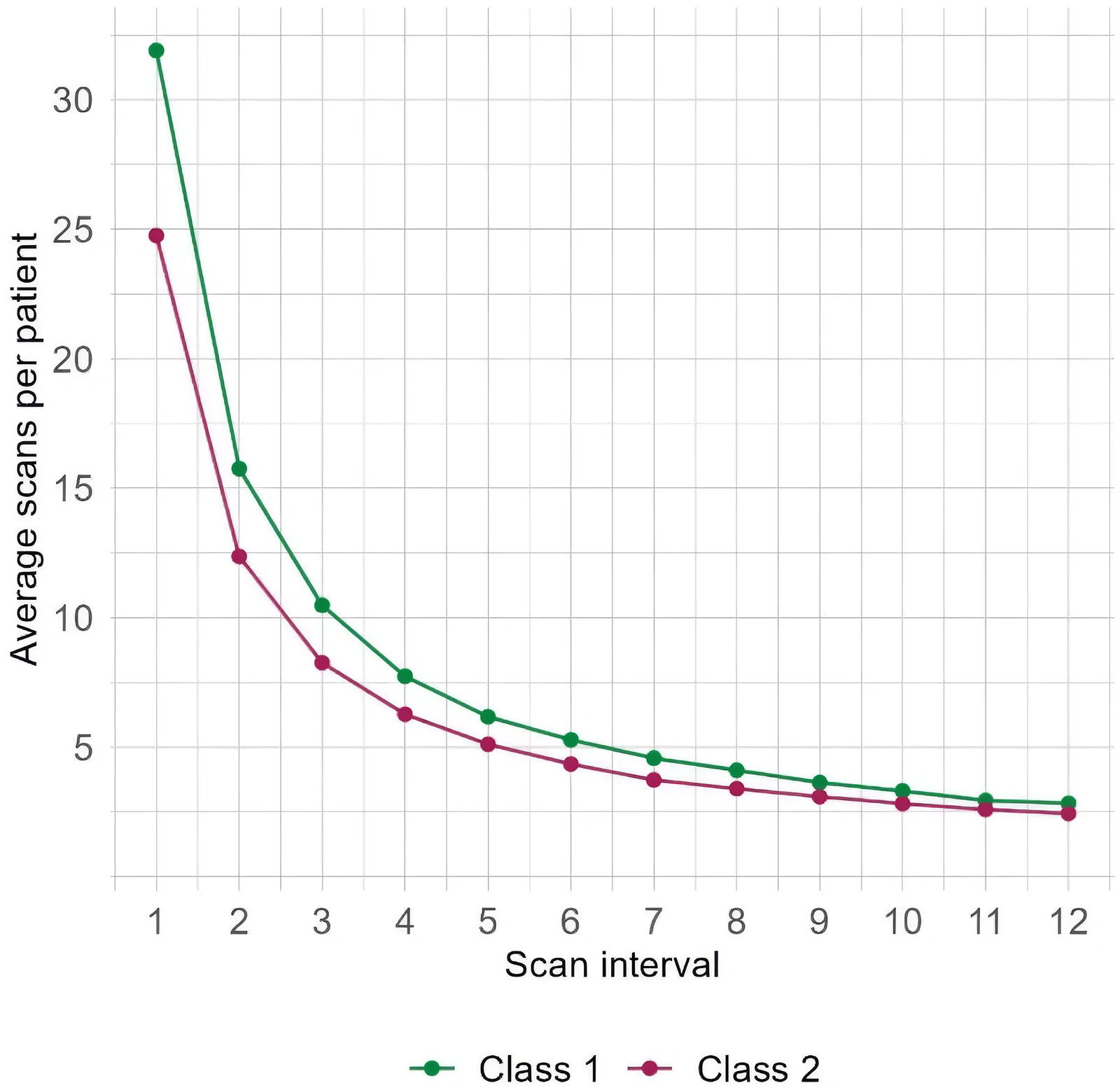
Average number of scans per patient by scan interval in all patients by class.

**Fig 4:**
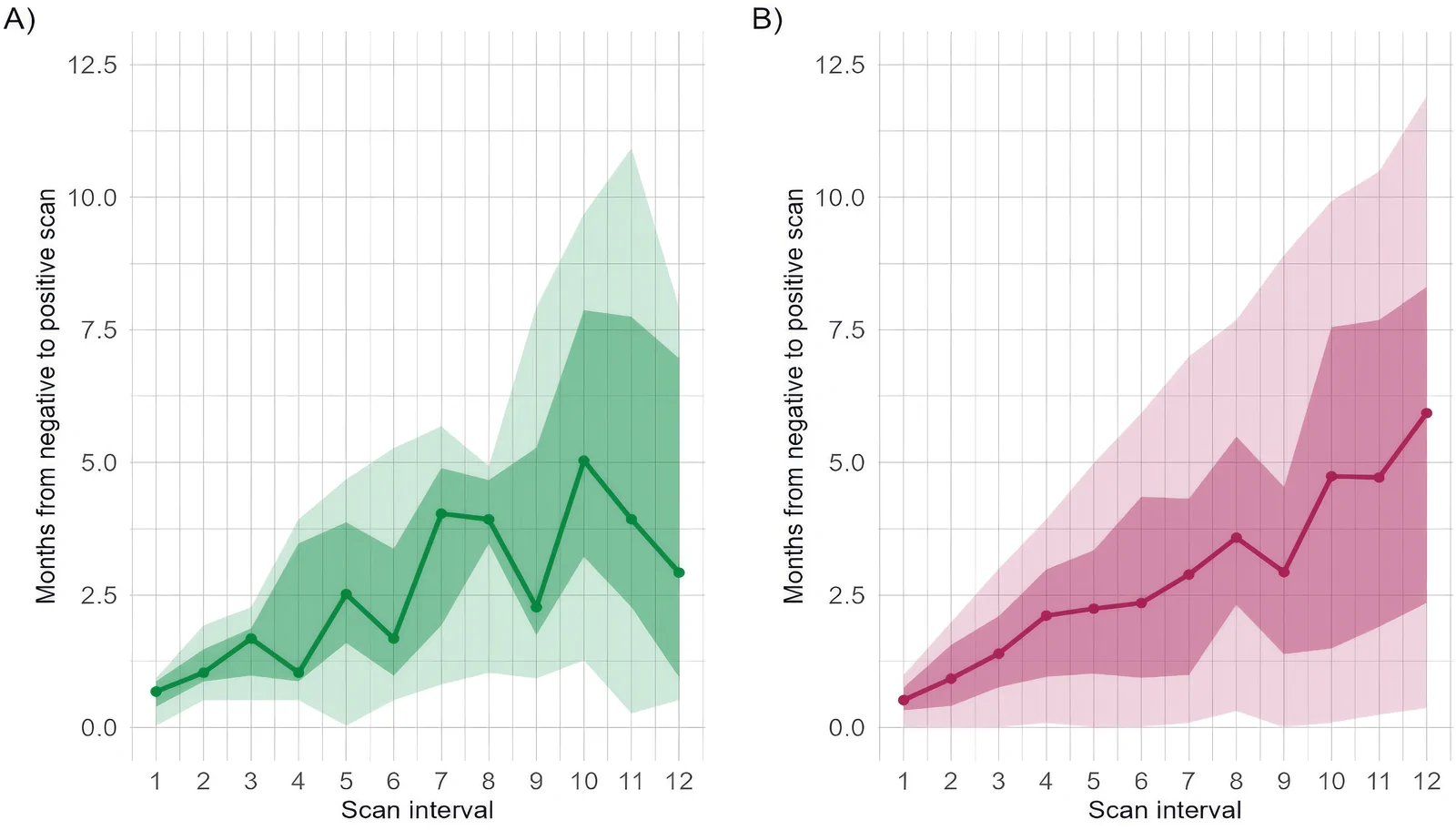
Months from hypothetical negative to observed positive scan by scan interval among patients who developed metastasis in Class 1 (A) and Class 2 (B) tumors.

When we stratify by Class, we find that in a hypothetical surveillance schedule, Class 2 patients have fewer average scans per patient as compared to Class 1 patients (Fig 3). However, the average months from negative to positive scan are similar between two Classes of patients (Fig 4A, 4B). Note that few Class 1 patients in these data developed metastasis, so that the results among patients with metastasis are based on only 7 Class 1 patients but on 60 Class 2 patients.

#### Class 1 patients.

ROC analysis for Class 1 patients showed that the 12-month scan interval minimized the Euclidean distance to the point (0, 0) with an average of 2.83 scans per patient and a median of 2.92 months from negative to positive scan. The 6-month scan interval had an average of 5.3 and a median from negative to positive scan of 2.35 months.

#### Class 2 patients.

ROC analysis for Class 2 patients showed that the 9-month scan interval minimized the Euclidean distance to the point (0, 0) with an average of 3.09 scans per patient and a median of 2.93 months from negative to positive scans. The 6-month scan interval had an average of 4.3 scans per patient and a median from negative to positive scan of 2.35 months

### Largest metastasis size and number of metastasis

The association between the scan interval and largest metastatic lesion and number of metastatic lesions was significant (p = 0.002) such that the largest metastasis lesion was significant larger when the scan interval was >=6 month versus <6 month. While not statistically significant, we also see numerically larger frequency of patients with>=5 metastasis lesions among patients with a >=6 month scan interval versus a < 6 months scan interval (**S3 Table**).

The association between the scan interval and largest metastatic lesion and number of metastatic lesions in Class 1 patients was underpowered as only 7 patients developed metastasis. In Class 2 patients, there is a significant association between largest metastatic lesion and observed scan interval, such that the largest metastatic lesion was significant larger when the scan interval was >=6 month versus <6 month. While not statistically significant, we also see numerically larger frequency of patients with greater number of metastatic lesions (>=5) among patients with a >=6 month scan interval versus a < 6 month scan interval (**S4 Table**)

### Sensitivity analyses

ROC analysis on additional dataset of 60 patients with unknown prognostication status (Class X) showed the ideal scan interval to be 9 months with 3.78 months from negative to positive scan and an average of 4.55 scans per patient, which aligns with the overall results and those for Class 2 (**S5 Table)**. This finding could be driven by the matching that was done to identify a subset of Class X patients for this sensitivity analysis, which resulted in a higher proportion of “Class 2-like” patients than “Class 1-like” patients.

## Discussion

In the present study we have limited analysis to the frequency of scans (interval between the scans) rather than modality (US, CT, MRI) because in our previous study, high frequency surveillance protocol (every 3 months) was at least equal if not superior to enhanced modality protocol (inclusion of CT/MRI) in detecting smaller metastatic lesions in high-risk patients [11].

Time since prior negative scan, defined as interval between a previous negative scan and the first positive scan was optimized as a surrogate for frequency of liver imaging. In this simplified novel approach for optimization, we performed statistical analyses using hypothetical surveillance schedules anchored in the real-world data of our patient cohort with known prognostication status. Hypothetical imaging frequencies ranging from every 1 month to every 12 months were explored.

For Class 1 patients, a 12-month scan interval was optimal as it had the minimal Euclidean distance to the point (0, 0) with a median of 2.92 months from negative to positive scan with an average of 2.83 scans per patient. Increasing the frequency to every 6 months, only modestly reduced the median scan interval from negative to positive scan by 17 days (2.92–2.35 months) while almost doubling the burden of scans per patients (5.5 vs 2.8 scans). For Class 2 patients,

A 9-month scan interval was optimal as it minimized the Euclidean distance to the point (0, 0) with an average of 3.09 scans per patients and a median of 2.93 months from negative to positive scans. Increasing the frequency to every 6 months only modestly reduced the median scan interval from negative to positive scan by 17 days (2.93–2.35 months) while increasing the burden of scans per patient by 25% (4.3 vs 3.09 scans). Although, the optimal scan interval of 9 months in those with unknown prognostication status aligned with those for Class 2, the small number of patients in Class XX (60) with fewer metastatic events (8) calls for cautious interpretation of the data.

The scan intervals seem to be clinically relevant as there was an association between the longer scan interval and size of metastatic lesion (>=6 months versus <6 months, p = 0.002). Recently published data from clinical trials for treatment of metastatic UM strongly supports the need for the detection of smaller hepatic metastatic lesions. In a randomized controlled trial, patients treated with tebentafusp-tebn had an improved OS (median 21.7 months [95% CI, 18.6–23.6]) compared with the investigator’s choice (median 16.0 months [95% CI, 9.7–19.4] in previously untreated HLAA* 02:01 positive patients (HR:0.51, 95% CI: 0.37, 0.71 p < 0.001) [32]. The improvement in OS was predominantly observed in those with smaller hepatic metastatic lesions (LDLM ≤ 3 cm [M1a]) (HR:0.36, 95% CI: 0.21, 0.61) and not in those with larger lesions (LDLM >3 cm) (HR:0.71, 95% CI: 0.44, 1.17 in M1b and HR:0.76, 95% CI: 0.34, 1.82 in M1c) [33]. Similarly, in the phase III (SCANDIUM Trial) trial the treatment benefit of isolated hepatic perfusion with melphalan was evident only in smaller hepatic metastatic lesions (LDLM ≤ 3 cm [M1a]) (HR: 0.46, 95% CI: 0.22, 0.95) and not in those with larger lesions (LDLM >3 cm) (HR:1.44, 95% CI: 0.57, 3.67 in M1b and HR:1.57, 95% CI: 0.22, 11.34 in M1c) [34,35]. The choice of surveillance protocols for the detection of metastasis may therefore influence not only the outcomes of a treatment trial as illustrated above but also those of adjuvant and neoadjuvant therapy trials [34,36–38].

No matter which modality or frequency is incorporated into the surveillance protocol, as there are reported differences in practice patterns [25,26], it should be modified over time in all patients [4,23] more so in high risk cases [7] (conditional survival) [39]. Among institutional/ national guidelines only those from Polish Society of Oncology [13] and NCCN seems to reflect that [40].

In addition to optimizing surveillance protocol, improvements in prognostication, i.e., identifying patients who are not at risk of metastasis would be beneficial so that such patients can be altogether excluded from surveillance. Such exclusion would apply to the majority of UM patients (69% have Class 1 tumors) [7] and about 95% of them have no short-term (5 year) risk of metastasis with incorporation of tumor diameter and PRAME status [7,10]. Other prognostication methods can also identify patients with low risk of such as those based on American Joint Committee on Cancer (AJCC) [41], tumors with disomy 3 chromosome, and EIF1AX mutations [42], and Liverpool Uveal Melanoma Prognosticator Online (LUMPO) [17]. Relative sensitivity and specificity of different prognostic systems, is discussed elsewhere [29]. Newer prognostication tools based upon circulating tumor DNA may further enhance risk prediction [43].

This study has several potential limitations, including its retrospective nature. To minimize sampling bias, only consecutive cases were considered for analysis and those who did not follow any specific protocol were excluded. Additionally, the study analysis is biased towards high-risk patients (Class 2) as metastatic events were low in Class 1 patients. Tumor factors such as location and tumor size, well known to modify the predicted risk, should be considered when personalizing the recommendations for an individual patient [44].

Our study, working within the larger paradigm of personalized medicine should be considered preliminary work that provides a frame-work for future studies that could refine our analysis.

**Key points**

Question: What is the optimal surveillance strategy for patients with uveal melanoma

Findings: For Class 1 patients (gene expression profile, GEP), 12-month scan interval was optimal with a median of 2.92 months from negative to positive scan with an average of 2.83 scans per patient. For Class 2 patients (GEP) 9-month scan interval was optimal with an average of 3.09 scans per patients and a median of 2.93 months from negative to positive scans.

Meaning: These analyses provide evidence-based guidance for developing risk-stratified surveillance strategy.

## Supporting information

S1 Table S1 Table. (DOCX)

S2 Table S2 Table. (DOCX)

S3 Table S3 Table. (DOCX)

S4 Table S4 Table. (DOCX)

S5 Table S5 Table. (DOCX)

S6 File Singh-optimal-uveal-surveillance UPLOADED. (CSV)

## Abbreviations

US: ultrasonography
OS: overall survival
UM: uveal melanoma
LDLM: largest diameter of largest hepatic metastasis
TDM: time to detection of metastasis
SP: standard protocol
EP: enhanced protocol
EM: enhanced modality
GEP: gene expression profile
