# Supplementary material for: Systemic surveillance for uveal melanoma: Approach to optimization
Source: PLoS One. 2026 Aug 19;21(8):e0355405. doi: 10.1371/journal.pone.0355405 (PMC13489437; doi:10.1371/journal.pone.0355405)
Supplement: Supplementary file 1 [file pone.0355405.s001.docx]

**Table S1. Patient and disease characteristics.**

| **Characteristic** | **N = 349^1^** | |
| --- | --- | --- |
| Age (years) | 62 (52, 69) | |
| Sex |  | |
| Female | 161 (46%) | |
| Male | 188 (54%) | |
| Laterality |  | |
| Right eye | 174 (50%) | |
| Left eye | 175 (50%) | |
| Location |  | |
| Iris | 22 (6.3%) | |
| Choroid | 262 (75%) | |
| Ciliary +/- Iris or Choroid | 65 (19%) | |
| Tumor size (mm) | 12.0 (9.5, 15.5) | |
| Tumor thickness (mm) | 4.4 (2.7, 7.6) | |
| GEP Class 1 | 227 | |
|  | Plaque therapy | 178 (78%) |
|  | Enucleation | 49 (22%) |
| GEP Class 2 | 122 | |
|  | Plaque therapy | 78 (64%) |
|  | Enucleation | 44 (36%) |
| PRAME status |  | |
| Negative | 185 (69%) | |
| Positive | 85 (31%) | |
| Unknown | 79 | |
| Follow-up (months) | 30.9 (19 - 57.9) | |
| Survival Status |  | |
| Dead from any cause | 48 (13%) | |
| Number with metastasis | 67 (19% | |
| ^1^Median (Q1, Q3); n (%) | | |

GEP: Gene expression profile
