# Supplementary material for: Systemic surveillance for uveal melanoma: Approach to optimization
Source: PLoS One. 2026 Aug 19;21(8):e0355405. doi: 10.1371/journal.pone.0355405 (PMC13489437; doi:10.1371/journal.pone.0355405)
Supplement: Supplementary file 2 [file pone.0355405.s002.docx]

Table S2. 5-year overall survival (OS) and metastasis-free survival (MFS) estimates,

overall and by GEP Class.

| **Characteristic** | **5-year OS** | **5-year MFS** |
| --- | --- | --- |
| Overall | 80% (74%, 86%) | 71% (65%, 78%) |
| GEP Class |  |  |
| Class 1 | 92% (87%, 98%) | 94% (90%, 99%) |
| Class 2 | 58% (47%, 73%) | 35% (25%, 49%) |

GEP: Gene expression profile
