# Supplementary material for: Systemic surveillance for uveal melanoma: Approach to optimization
Source: PLoS One. 2026 Aug 19;21(8):e0355405. doi: 10.1371/journal.pone.0355405 (PMC13489437; doi:10.1371/journal.pone.0355405)
Supplement: Supplementary file 3 [file pone.0355405.s003.docx]

**Table S3. Metastasis size and number, by months from negative to positive scan**

| **Characteristic** | **<6-month**  **N = 39^1^** | **>=6-month**  **N = 28^1^** | **p-value^2^** |
| --- | --- | --- | --- |
| Largest met lesion (cm) | 1.70 (1.10, 2.50) | 3.75 (1.45, 5.90) | 0.002 |
| Number of met lesions |  |  | 0.077 |
| 1 | 11 (28%) | 2 (7.1%) |  |
| 2 | 5 (13%) | 1 (3.6%) |  |
| 3 | 3 (7.7%) | 2 (7.1%) |  |
| 4 | 3 (7.7%) | 2 (7.1%) |  |
| >=5 | 17 (44%) | 21 (75%) |  |
| ^1^Median (Q1, Q3); n (%) | | | |
| ^2^Wilcoxon rank sum test; Fisher's exact test | | | |
|  | | | |
