# Supplementary material for: Systemic surveillance for uveal melanoma: Approach to optimization
Source: PLoS One. 2026 Aug 19;21(8):e0355405. doi: 10.1371/journal.pone.0355405 (PMC13489437; doi:10.1371/journal.pone.0355405)
Supplement: Supplementary file 4 [file pone.0355405.s004.docx]

**Table S4. Metastasis size and number, by months from negative to positive scan (By GEP Class)**

| **Characteristic** | **Class 1** | | | | **Class 2** | | |
| --- | --- | --- | --- | --- | --- | --- | --- |
|  | **<6-month**  **N = 39^1^** | **<6-month**  **N = 4^1^** | **>=6-month**  **N = 3^1^** | **p-value^3^** | **<6-month**  **N = 35^1^** | **>=6-month**  **N = 25^1^** | **p-value^2^** |
| Largest met lesion (cm) | 1.70  (1.10, 2.50) | 2.70  (1.70, 5.25) | 3.80  (1.50, 5.10) | >0.9 | 1.60  (1.00, 2.50) | 3.70  (1.40, 6.10) | 0.002 |
| Number of met lesions |  |  |  | 0.4 |  |  | 0.12 |
| 1 | 11 (28%) | 2 (50%) | 0 (0%) |  | 9 (26%) | 2 (8.0%) |  |
| 2 | 5 (13%) | 0 (0%) | 0 (0%) |  | 5 (14%) | 1 (4.0%) |  |
| 3 | 3 (7.7%) | 0 (0%) | 0 (0%) |  | 3 (8.6%) | 2 (8.0%) |  |
| 4 | 3 (7.7%) | 0 (0%) | 1 (33%) |  | 3 (8.6%) | 1 (4.0%) |  |
| >=5 | 17 (44%) | 2 (50%) | 2 (67%) |  | 15 (43%) | 19 (76%) |  |
| ^1^Median (Q1, Q3); n (%) | | | | | | |  |
| ^2^Wilcoxon rank sum test; Fisher's exact test | | | | | | |  |
| ^3^Wilcoxon rank sum exact test; Fisher's exact test | | | | | | |  |
