# Supplementary material for: Systemic surveillance for uveal melanoma: Approach to optimization
Source: PLoS One. 2026 Aug 19;21(8):e0355405. doi: 10.1371/journal.pone.0355405 (PMC13489437; doi:10.1371/journal.pone.0355405)
Supplement: Supplementary file 5 [file pone.0355405.s005.docx]

**Table S5. Overall results and months from negative to positive scan by scan interval,**

**among those with metastasis (Class X).**

| **Scan interval**  **(months)** | **Average scans/**  **patient** | **Interval**  **Negative to Positive Scan** | | |
| --- | --- | --- | --- | --- |
|  |  | **Median** | **(Min, Max)** | **(Q1, Q3)** |
| 1 | 43.9 | 0.60 | (0.13, 0.93) | (0.39, 0.74) |
| 2 | 21.8 | 1.24 | (0.40, 1.93) | (0.76, 1.58) |
| 3 | 14.4 | 2.24 | (0.40, 2.93) | (1.42, 2.60) |
| 4 | 10.7 | 2.93 | (0.76, 3.93) | (2.14, 3.58) |
| 5 | 8.5 | 2.74 | (1.64, 3.74) | (1.89, 3.44) |
| 6 | 7.0 | 3.93 | (0.40, 5.93) | (1.42, 5.40) |
| 7 | 5.9 | 3.49 | (1.76, 4.93) | (2.45, 3.90) |
| 8 | 5.2 | 5.75 | (1.35, 7.64) | (3.54, 7.24) |
| 9 | 4.5 | 3.78 | (0.40, 8.35) | (2.24, 5.24) |
| 10 | 4.3 | 5.16 | (1.64, 8.74) | (2.83, 7.61) |
| 11 | 3.8 | 4.75 | (0.93, 9.64) | (1.45, 6.61) |
| 12 | 3.6 | 6.40 | (0.76, 11.56) | (4.04, 9.38) |
