# Supplementary material for: Systemic surveillance for uveal melanoma: Approach to optimization
Source: PLoS One. 2026 Aug 19;21(8):e0355405. doi: 10.1371/journal.pone.0355405 (PMC13489437; doi:10.1371/journal.pone.0355405)
Supplement: Supplementary file 6 [file pone.0355405.s007.docx]

PONE-D-26-09309

Systemic surveillance for uveal melanoma: Approach to optimization

PLOS One
Vikas Khetan, MD
Academic Editor
PLOS One

Thank you for considering our manuscript.

Reviewer #1: The authors propose tailoring follow-up regimens for patients with uveal melanoma and successfully derive cutoffs for screening intervals using hypothetical surveillance schedules for low-risk and high-risk cohorts. The work is very relevant to the management of uveal melanoma in ocular oncology practice.

Thank you

A few comments to improve the manuscript:
1. Key points: Please mention “GEP Class 1” and “GEP Class 2”

ADDED
2. Abstract: “
Suggest rephrasing this as follows, for clarity:
“Our analysis suggests that the frequency of hepatic imaging for detection of metastasis can be tailored based on the risk status to 12 months and 9 months for patients with low and high-risk tumors, respectively.”

DONE

3. Results - Demographics: Please modify this to: “The median patient age was 62 years, and 54% were male.”

DONE

4. Results – Sensitivity analyses: Interestingly, the screening interval for the cohort with unknown risk status corresponds to that derived for the high-risk cohort. Are the authors able to review and identify any clinic-pathological features in this cohort that favor a GEP Class 2 profile, to explain this finding? Please discuss this finding as it can aid in the management of patients who refuse molecular prognostication/ those with tumors not amenable to biopsy.

ADDED IN Methods, Results and discussion

Methods
Sensitivity analysis was performed in a separate cohort of patients with unknown risk (Class X). To identify a subset of Class X patients to conduct sensitivity analysis, we matched Class X patients to patients with known class to ensure patients with a variety of features were included. Matching was done using nearest neighbor matching based on tumor location and thickness. In this way, of the original 169 Class X eyes, 40 were matched to Class 2 patients and 20 were matched to Class 1 patients.

Results

ROC analysis on additional dataset of 60 patients with unknown prognostication status (Class X) showed the ideal scan interval to be 9 months with 3.78 months from negative to positive scan and an average of 4.55 scans per patient, which aligns with the overall results and those for Class 2 (Supplement Table E). This finding could be driven by the matching that was done to identify a subset of Class X patients for this sensitivity analysis, which resulted in a higher proportion of “Class 2-like” patients than “Class 1-like” patients.

Discussion

Although, the optimal scan interval of 9 months in those with unknown prognostication status aligned with the overall results and those for Class 2, the small number of patients in Class XX (60) with fewer metastatic events (8) calls for cautious interpretation of the data..

Reviewer #2: This is a great attempt to standardise surveillance schedules for UM

Thank you

Abstract - design - add “retrospective”
ADDED

Table 1 - ?? Not found

UPLOADED

Supplemental table A/S1 - how many from GEP 1 and GEP 2 underwent enucleation or plaque

ADDED in the table 1

| GEP Class 1 | 227 | |
| --- | --- | --- |
|  | Plaque therapy | 178 (78%) |
|  | Enucleation | 49 (22%) |
| GEP Class 2 | 122 | |
|  | Plaque therapy | 78 (64%) |
|  | Enucleation | 44 (36%) |

Any metastasis correlation with tumor size

The focus of this paper was on assessment of risk stratified surveillance protocols rather the risk stratification itself. Hence not included.
