# Supplementary material for: Systemic surveillance for uveal melanoma: Approach to optimization
Source: PLoS One. 2026 Aug 19;21(8):e0355405. doi: 10.1371/journal.pone.0355405 (PMC13489437; doi:10.1371/journal.pone.0355405)
Supplement: Peer review history

# Peer review: Decision Letter 0

**DOI:** 10.1371/journal.pone.0355405.r001

**Authors:** Vikas Khetan

16 Jun 2026

Dear Dr. Singh,

Thank you for submitting your manuscript to PLOS ONE. After careful consideration, we feel that it has merit but does not fully meet PLOS ONE’s publication criteria as it currently stands. Therefore, we invite you to submit a revised version of the manuscript that addresses the points raised during the review process.

Please submit your revised manuscript by Jul 31 2026 11:59PM. If you will need more time than this to complete your revisions, please reply to this message or contact the journal office at plosone@plos.org. When you're ready to submit your revision, log on to https://www.editorialmanager.com/pone/ and select the 'Submissions Needing Revision' folder to locate your manuscript file.

- A letter that responds to each point raised by the academic editor and reviewer(s). You should upload this letter as a separate file labeled 'Response to Reviewers'.
- A marked-up copy of your manuscript that highlights changes made to the original version. You should upload this as a separate file labeled 'Revised Manuscript with Track Changes'.
- An unmarked version of your revised paper without tracked changes. You should upload this as a separate file labeled 'Manuscript'.

If you would like to make changes to your financial disclosure, please include your updated statement in your cover letter. Guidelines for resubmitting your figure files are available below the reviewer comments at the end of this letter.

If applicable, we recommend that you deposit your laboratory protocols in protocols.io to enhance the reproducibility of your results. Protocols.io assigns your protocol its own identifier (DOI) so that it can be cited independently in the future. For instructions see: https://journals.plos.org/plosone/s/submission-guidelines#loc-laboratory-protocols. Additionally, PLOS ONE offers an option for publishing peer-reviewed Lab Protocol articles, which describe protocols hosted on protocols.io. Read more information on sharing protocols at https://plos.org/protocols?utm_medium=editorial-email&utm_source=authorletters&utm_campaign=protocols.

As the corresponding author, your ORCID iD is verified in the submission system and will appear in the published article. PLOS supports the use of ORCID, and we encourage all coauthors to register for an ORCID iD and use it as well. Please encourage your coauthors to verify their ORCID iD within the submission system before final acceptance, as unverified ORCID iDs will not appear in the published article. *Only*  the individual author can complete the verification step; PLOS staff *cannot*  verify ORCID iDs on behalf of authors.

We look forward to receiving your revised manuscript.

Kind regards,

Vikas Khetan, MD

Academic Editor

PLOS One

Journal Requirements:

When submitting your revision, we need you to address these additional requirements.

1. Please ensure that your manuscript meets PLOS ONE's style requirements, including those for file naming. The PLOS ONE style templates can be found at

https://journals.plos.org/plosone/s/file?id=wjVg/PLOSOne_formatting_sample_main_body.pdf and

https://journals.plos.org/plosone/s/file?id=ba62/PLOSOne_formatting_sample_title_authors_affiliations.pdf

2. Please include captions for your Supporting Information files at the end of your manuscript, and update any in-text citations to match accordingly. Please see our Supporting Information guidelines for more information: http://journals.plos.org/plosone/s/supporting-information.

3. If the reviewer comments include a recommendation to cite specific previously published works, please review and evaluate these publications to determine whether they are relevant and should be cited. There is no requirement to cite these works unless the editor has indicated otherwise.

4. Please review your reference list to ensure that it is complete and correct. If you have cited papers that have been retracted, please include the rationale for doing so in the manuscript text, or remove these references and replace them with relevant current references. Any changes to the reference list should be mentioned in the rebuttal letter that accompanies your revised manuscript. If you need to cite a retracted article, indicate the article’s retracted status in the References list and also include a citation and full reference for the retraction notice.

[Note: HTML markup is below. Please do not edit.]

Reviewers' comments:

Reviewer's Responses to Questions

**Comments to the Author**

1. Is the manuscript technically sound, and do the data support the conclusions?

Reviewer #1: Yes

Reviewer #2: Yes

**********

2. Has the statistical analysis been performed appropriately and rigorously?

Reviewer #1: Yes

Reviewer #2: Yes

**********

3. Have the authors made all data underlying the findings in their manuscript fully available?

The PLOS Data policy

Reviewer #1: Yes

Reviewer #2: Yes

**********

4. Is the manuscript presented in an intelligible fashion and written in standard English?

Reviewer #1: Yes

Reviewer #2: Yes

**********

Reviewer #1: The authors propose tailoring follow-up regimens for patients with uveal melanoma and successfully derive cutoffs for screening intervals using hypothetical surveillance schedules for low-risk and high-risk cohorts. The work is very relevant to the management of uveal melanoma in ocular oncology practice.

A few comments to improve the manuscript:

1. Key points: Please mention “GEP Class 1” and “GEP Class 2”

2. Abstract: “Our analysis suggests that optimal frequency of hepatic imaging for detection of metastasis is prognostication status dependent ranging from 9 months to 12 months for patients with low and high-risk tumors, respectively.”

Suggest rephrasing this as follows, for clarity:

“Our analysis suggests that the frequency of hepatic imaging for detection of metastasis can be tailored based on the risk status to 12 months and 9 months for patients with low and high-risk tumors, respectively.”

3. Results - Demographics: “Median patient age was 62 and 54% were male.”

Please modify this to: “The median patient age was 62 years, and 54% were male.”

4. Results – Sensitivity analyses: Interestingly, the screening interval for the cohort with unknown risk status corresponds to that derived for the high-risk cohort. Are the authors able to review and identify any clinic-pathological features in this cohort that favor a GEP Class 2 profile, to explain this finding? Please discuss this finding as it can aid in the management of patients who refuse molecular prognostication/ those with tumors not amenable to biopsy.

Reviewer #2: This is a great attempt to standardise surveillance schedules for UM

Abstract - design - add “retrospective”

Table 1 - ?? Not found

Supplemental table A/S1 - how many from GEP 1 and GEP 2 underwent enucleation or plaque

Any metastasis correlation with tumour size

**********

what does this mean?). If published, this will include your full peer review and any attached files.

If you choose “no”, your identity will remain anonymous but your review may still be made public.

**Do you want your identity to be public for this peer review?**  For information about this choice, including consent withdrawal, please see our Privacy Policy

Reviewer #1: No

Reviewer #2: No

**********

[NOTE: If reviewer comments were submitted as an attachment file, they will be attached to this email and accessible via the submission site. Please log into your account, locate the manuscript record, and check for the action link "View Attachments". If this link does not appear, there are no attachment files.]

To ensure your figures meet our technical requirements, please review our figure guidelines: https://journals.plos.org/plosone/s/figures

You may also use PLOS’s free figure tool, NAAS, to help you prepare publication quality figures: https://journals.plos.org/plosone/s/figures#loc-tools-for-figure-preparation.

NAAS will assess whether your figures meet our technical requirements by comparing each figure against our figure specifications.

# Author comment: Author response to Decision Letter 1

**DOI:** 10.1371/journal.pone.0355405.r002

2 Jul 2026

PONE-D-26-09309

Systemic surveillance for uveal melanoma: Approach to optimization

PLOS One

Vikas Khetan, MD

Academic Editor

PLOS One

Thank you for considering our manuscript.

Reviewer #1: The authors propose tailoring follow-up regimens for patients with uveal melanoma and successfully derive cutoffs for screening intervals using hypothetical surveillance schedules for low-risk and high-risk cohorts. The work is very relevant to the management of uveal melanoma in ocular oncology practice.

Thank you

A few comments to improve the manuscript:

1. Key points: Please mention “GEP Class 1” and “GEP Class 2”

ADDED

2. Abstract: “

Suggest rephrasing this as follows, for clarity:

“Our analysis suggests that the frequency of hepatic imaging for detection of metastasis can be tailored based on the risk status to 12 months and 9 months for patients with low and high-risk tumors, respectively.”

DONE

3. Results - Demographics: Please modify this to: “The median patient age was 62 years, and 54% were male.”

DONE

4. Results – Sensitivity analyses: Interestingly, the screening interval for the cohort with unknown risk status corresponds to that derived for the high-risk cohort. Are the authors able to review and identify any clinic-pathological features in this cohort that favor a GEP Class 2 profile, to explain this finding? Please discuss this finding as it can aid in the management of patients who refuse molecular prognostication/ those with tumors not amenable to biopsy.

ADDED IN Methods, Results and discussion

Methods

Sensitivity analysis was performed in a separate cohort of patients with unknown risk (Class X). To identify a subset of Class X patients to conduct sensitivity analysis, we matched Class X patients to patients with known class to ensure patients with a variety of features were included. Matching was done using nearest neighbor matching based on tumor location and thickness. In this way, of the original 169 Class X eyes, 40 were matched to Class 2 patients and 20 were matched to Class 1 patients.

Results

ROC analysis on additional dataset of 60 patients with unknown prognostication status (Class X) showed the ideal scan interval to be 9 months with 3.78 months from negative to positive scan and an average of 4.55 scans per patient, which aligns with the overall results and those for Class 2 (Supplement Table E). This finding could be driven by the matching that was done to identify a subset of Class X patients for this sensitivity analysis, which resulted in a higher proportion of “Class 2-like” patients than “Class 1-like” patients.

Discussion

Although, the optimal scan interval of 9 months in those with unknown prognostication status aligned with the overall results and those for Class 2, the small number of patients in Class XX (60) with fewer metastatic events (8) calls for cautious interpretation of the data..

Reviewer #2: This is a great attempt to standardise surveillance schedules for UM

Thank you

Abstract - design - add “retrospective”

ADDED

Table 1 - ?? Not found

UPLOADED

Supplemental table A/S1 - how many from GEP 1 and GEP 2 underwent enucleation or plaque

ADDED in the table 1

GEP Class 1

227

Plaque therapy 178 (78%)

Enucleation 49 (22%)

GEP Class 2

122

Plaque therapy 78 (64%)

Enucleation 44 (36%)

Any metastasis correlation with tumor size

The focus of this paper was on assessment of risk stratified surveillance protocols rather the risk stratification itself. Hence not included.

Attachment Submitted filename: PONE REVEIW.docx

# Peer review: Decision Letter 1

**DOI:** 10.1371/journal.pone.0355405.r003

**Authors:** Vikas Khetan

21 Jul 2026

Systemic surveillance for uveal melanoma: Approach to optimization

PONE-D-26-09309R1

Dear Dr. Singh,

We’re pleased to inform you that your manuscript has been judged scientifically suitable for publication and will be formally accepted for publication once it meets all outstanding technical requirements.

Within one week, you’ll receive an e-mail detailing the required amendments. When these have been addressed, you’ll receive a formal acceptance letter and your manuscript will be scheduled for publication.

An invoice will be generated when your article is formally accepted. Please note, if your institution has a publishing partnership with PLOS and your article meets the relevant criteria, all or part of your publication costs will be covered. Please make sure your user information is up-to-date by logging into Editorial Manager at Editorial Manager® and clicking the ‘Update My Information' link at the top of the page. For questions related to billing, please contact billing support.

If your institution or institutions have a press office, please notify them about your upcoming paper to help maximize its impact. If they’ll be preparing press materials, please inform our press team as soon as possible -- no later than 48 hours after receiving the formal acceptance. Your manuscript will remain under strict press embargo until 2 pm Eastern Time on the date of publication. For more information, please contact onepress@plos.org.

Kind regards,

Vikas Khetan, MD

Academic Editor

PLOS One

Additional Editor Comments (optional):

Reviewers' comments:

Reviewer's Responses to Questions

**Comments to the Author**

Reviewer #1: All comments have been addressed

Reviewer #2: All comments have been addressed

**********

2. Is the manuscript technically sound, and do the data support the conclusions?

Reviewer #1: Yes

Reviewer #2: Yes

**********

3. Has the statistical analysis been performed appropriately and rigorously?

Reviewer #1: Yes

Reviewer #2: Yes

**********

4. Have the authors made all data underlying the findings in their manuscript fully available?

The PLOS Data policy

Reviewer #1: Yes

Reviewer #2: Yes

**********

5. Is the manuscript presented in an intelligible fashion and written in standard English?

Reviewer #1: Yes

Reviewer #2: Yes

**********

Reviewer #1: Thanks for addressing all the suggestions and comments.

The paper is very much relevant in the current era.

Best of luck for the paper.

Reviewer #2: All comments in the previous draft have been answered. The manuscript has been updated accordingly. No further comments

**********

what does this mean?). If published, this will include your full peer review and any attached files.

If you choose “no”, your identity will remain anonymous but your review may still be made public.

**Do you want your identity to be public for this peer review?**  For information about this choice, including consent withdrawal, please see our Privacy Policy

Reviewer #1: No

Reviewer #2: No

**********

# Editor report: Acceptance letter

**DOI:** 10.1371/journal.pone.0355405.r004

**Authors:** Vikas Khetan

PONE-D-26-09309R1

PLOS One

Dear Dr. Singh,

I'm pleased to inform you that your manuscript has been deemed suitable for publication in PLOS One. Congratulations! Your manuscript is now being handed over to our production team.

At this stage, our production department will prepare your paper for publication. This includes ensuring the following:

* All references, tables, and figures are properly cited

* All relevant supporting information is included in the manuscript submission,

* There are no issues that prevent the paper from being properly typeset

You will receive further instructions from the production team, including instructions on how to review your proof when it is ready. Please keep in mind that we are working through a large volume of accepted articles, so please give us a few days to review your paper and let you know the next and final steps.

Lastly, if your institution or institutions have a press office, please let them know about your upcoming paper now to help maximize its impact. If they'll be preparing press materials, please inform our press team within the next 48 hours. Your manuscript will remain under strict press embargo until 2 pm Eastern Time on the date of publication. For more information, please contact onepress@plos.org.

You will receive an invoice from PLOS for your publication fee after your manuscript has reached the completed accept phase. If you receive an email requesting payment before acceptance or for any other service, this may be a phishing scheme. Learn how to identify phishing emails and protect your accounts at https://explore.plos.org/phishing.

If we can help with anything else, please email us at customercare@plos.org.

Thank you for submitting your work to PLOS ONE and supporting open access.

Kind regards,

PLOS ONE Editorial Office Staff

on behalf of

Dr. Vikas Khetan

Academic Editor

PLOS One
